# Editorial: Pathogenesis, treatment, and future directions for rare T-cell leukemias

**DOI:** 10.3389/fonc.2022.991527

**Published:** 2022-09-07

**Authors:** Marco Herling, Wael Jarjour, Anjali Mishra, Jonathan E. Brammer

**Affiliations:** ^1^ Department of Hematology, Cellular Therapy, and Hemostasis University of Leipzig, Leipzig, Germany; ^2^ Department of Internal Medicine, Center for Integrated Oncology (CIO-ABCD), Aachen-Bonn-Cologne-Duesseldorf, Excellence Cluster for Cellular Stress Response and Aging-Associated Diseases (CECAD), Center for Molecular Medicine Cologne (CMMC), University of Cologne (UoC), Cologne, Germany; ^3^ Division of Rheumatology, The Ohio State University, Columbus, OH, United States; ^4^ Division of Hematologic Malignancies and Hematopoietic Stem Cell Transplantation, Department of Medical Oncology and Department of Cancer Biology, Sydney Kimmel Cancer Center, Thomas Jefferson University, Philadelphia, PA, United States; ^5^ Division of Hematology, Department of Internal Medicine, James Comprehensive Cancer Center, The Ohio State University, Columbus, OH, United States

**Keywords:** large granular lymphocyte (LGL) leukemia, T-prolymphocytic leukemia, cytokine, IL-15, T-cell leukemia, Mature T-cell leukemia

Mature T-cell leukemias represent rare, but increasingly recognized diseases of which, compared to their B-cell counterparts, comparatively little is established on their pathogenesis, diagnosis, and treatment. These leukemic post-thymic T-cell neoplasms range from the spectrum of chronic, sometimes debilitating disorders such as T-large granular lymphocytic leukemia (T-LGLL), and related leukemias such as NK-LGLL, to more aggressive malignancies such as T- prolymphocytic leukemia (T-PLL). In this series, entitled ‘Pathogenesis, Treatment, and Future Directions for Rare T-cell Leukemias’ we review the current state of the science of these important T-cell neoplasms to inform on their treatment, diagnosis, and pathophysiology.

First, in the review by El-Sharkawi et al., the diagnosis of T-cell leukemias is appraised in detail, with a practical guide to the spectrum of T-cell leukemias. Subsequently, the series can be divided between different reports on T-PLL and T-LGLL, with one paper by Yin et al., evaluating the prognostic importance of genomic mutations in patients with (immature) T-cell acute lymphoblastic leukemia.

Two papers review our current understanding of the pathogenesis and management of T-PLL. In the review by Braun et al., the authors summarize the known pathogenetic data of T-PLL and propose an intriguing model using the key molecular drivers of T-PLL to inform future translational approaches. In the second review by Varadarajan and Ballen, the authors describe the current state of cellular therapies, including allogeneic stem cell transplantation and emerging novel strategies to treat T-PLL that will guide clinicians as they seek to provide curative therapies for these patients.

A key focus of this series is on T-LGLL in which 7 papers, ranging from original data and cases series to cross-disciplinary reviews, provide a perspective on the current understanding of this disease. Drillet et al., review recent data on the diagnosis of NK-LGL, and provide a classification system that will likely serve as the standard for categorizing this rare leukemia for future investigations. Cytokines are integral in the biology of T-LGLL, and Isabelle et al., provide the most comprehensive review of cytokines and their contribution to T-LGLL pathogenesis to date. T-LGLL often overlaps with autoimmune disorders, such as rheumatoid arthritis (RA), hence, providing a fascinating opportunity to explore the intersection between cancer and autoimmunity, with important implications for the management of both. In the review by Couette et al., the authors evaluate the pathogenesis of T-LGLL, particularly as it relates to cytokines and key molecular pathways in a broad array of autoimmune diseases. In a focused review evaluating the intersection between RA and LGLL, Moosic et al., outline the current understanding of the mechanistic links between RA and LGLL. In two reports by Pflug et al., and Schreiber et al., the authors present illustrative case catalogues of T-LGLL, with a focus on diagnosis and cross-disciplinary management of these often complex patients, with a review of current treatment strategies. Finally, in an original report by Braunstein et al., the authors present the largest series of patients with concomitant plasma cell dyscrasias and T-cell malignancies, including T-LGLL to date, raising awareness of these co-incident disorders, with important recommendations on the management of these diseases.

This Research Topic represents the current state-of-the art understanding of mature T-cell leukemias, with a focus on T-PLL and T-LGLL. The knowledge gained from recent investigations into these diseases has led to increased interest not only amongst lab-based and clinical researchers, but also among pharmaceutical companies to address these rare malignancies. In T-PLL, this has manifested in the work of the T-PLL International Study Group (TPLL-ISG), that is leading the development of novel clinical trials based on the current understanding of the pathogenesis of T-PLL, as outlined in the review by Braun et al. This group has recently published consensus criteria on the diagnosis and treatment responses for this disease, an important step in developing trials for T-PLL (1). In fact, several trials are currently enrolling for patients with T-PLL and target the pathways described in the review by Braun et al., (NCT04496349, NCT03989466). Similarly, in T-LGLL, there has been renewed interest in developing novel therapeutics, given the modest efficacy of current immune-suppressive therapies. In particular, research has targeted the cytokine IL-15, as this is thought to be the central cytokine that drives the pathogenesis of T-LGL as was elegantly outlined in the reviews by Isabelle et al. and Couette et al. A recently completed phase I/II study utilized the selective cytokine inhibiting peptide BNZ-1 in T-LGLL patients, and reported clinical efficacy and near-universal apoptosis of *in vivo* T-LGLL cells, demonstrating the cytokine dependence of T-LGLL (2). Further, using an alternate approach targeting IL-15, the only currently enrolling prospective trial in the United States (NCT05141682) uses the hypomethylating agent CC-486 to treat patients with T-LGLL based on data demonstrating its efficacy in decreasing IL-15 (3). Using these approaches and others, we hope that significant progress can be made in treating this rare disease.

Finally, the editors wish to thank all who contributed to this important Research Topic. It is our sincere hope that this Research Topic will help to educate and inspire the development of innovative treatment approaches in these rare diseases that will impact patient outcomes.

## Author contributions

MH contributed to the development of the manuscript, revised the manuscript, and approved the final version WJ contributed to the development of the manuscript, revised the manuscript, and approved the final version AM contributed to the development of the manuscript, revised the manuscript, and approved the final version JB wrote the manuscript, revised the manuscript. All authors contributed to the article and approved the submitted version.

## Acknowledgments

Finally, the editors wish to thank all who contributed to this important series.

## Conflict of interest

The authors declare that the research was conducted in the absence of any commercial or financial relationships that could be construed as a potential conflict of interest.

## Publisher’s note

All claims expressed in this article are solely those of the authors and do not necessarily represent those of their affiliated organizations, or those of the publisher, the editors and the reviewers. Any product that may be evaluated in this article, or claim that may be made by its manufacturer, is not guaranteed or endorsed by the publisher.
